# Geometry Sampling-Based Adaption to DCGAN for 3D Face Generation †

**DOI:** 10.3390/s23041937

**Published:** 2023-02-09

**Authors:** Guoliang Luo, Guoming Xiong, Xiaojun Huang, Xin Zhao, Yang Tong, Qiang Chen, Zhiliang Zhu, Haopeng Lei, Juncong Lin

**Affiliations:** 1 Virtual Reality and Interactive Techniques Institute, East China Jiaotong University, Nanchang 330013, China; 2 School of Computer Science, Jiangxi Normal University, Nanchang 330022, China; 3 School of Information, Xiamen University, Xiamen 361005, China

**Keywords:** geometry sampling, 3D face generation, depth-like map sampling, structured representation, DCGAN

## Abstract

Despite progress in the past decades, 3D shape acquisition techniques are still a threshold for various 3D face-based applications and have therefore attracted extensive research. Moreover, advanced 2D data generation models based on deep networks may not be directly applicable to 3D objects because of the different dimensionality of 2D and 3D data. In this work, we propose two novel sampling methods to represent 3D faces as matrix-like structured data that can better fit deep networks, namely (1) a geometric sampling method for the structured representation of 3D faces based on the intersection of iso-geodesic curves and radial curves, and (2) a depth-like map sampling method using the average depth of grid cells on the front surface. The above sampling methods can bridge the gap between unstructured 3D face models and powerful deep networks for an unsupervised generative 3D face model. In particular, the above approaches can obtain the structured representation of 3D faces, which enables us to adapt the 3D faces to the Deep Convolution Generative Adversarial Network (DCGAN) for 3D face generation to obtain better 3D faces with different expressions. We demonstrated the effectiveness of our generative model by producing a large variety of 3D faces with different expressions using the two novel down-sampling methods mentioned above.

## 1. Introduction

With the rapid advancements of display equipments and growing network bandwidths, 3D data are becoming another popular media due to the fullness of realistic information. On the other hand, nowadays 3D shape acquisitions either rely on expensive equipment or require expert knowledge and skills. For this reason, 3D data acquisition techniques remain a threshold for the broader application of 3D data.

3D shape synthesis techniques provide an alternative means for 3D data generation, which can be useful for various purposes without copyright infringement [1,2]. Specifically, 3D synthetic faces can be broadly used in video games, beauty applications, virtual reality and so on. However, although various synthetic approaches for 2D images and video have been intensively studied, 3D shape synthesis remains a challenging task for the following reasons: (1) A 3D shape is a set of 3D vertices in the space, comprising un-structured data, compared to 2D images that can be stored in a structured 2D matrix. (2) A 3D shape is sensitive to noises, while one or even a few outlier pixels may not be visually noticeable in a 2D image.

This work is an extension of our published paper [3], the contributions are two-fold:

- First, we propose two new sampling methods to represent 3D faces as a structured matrix, which enables us to generate 3D shapes with deep networks. Specifically, they are the geometry sampling method and the depth map-like sampling method.
- Second, we present a straight-forward unsupervised 3D face generative model, which does not require any pre-processing steps such as the extraction of facial feature points or pre-computing the correspondences.

The remainder of the paper is organized as follows. We first briefly review the state-of-the-art in Section 2. Then, we present our geometric 3D face generation model in Section 3, followed by results and discussions in Section 4. Finally, we conclude the work in Section 5.

## 2. Related Work

Although Deep Neural Network (DNN)-based data-driven synthetic methods have been intensively studied in the computer vision field [4], it remains a challenging topic for data-driven 3D shape generation. In this section, we first briefly review the 3D shape reconstruction techniques in the computer vision domain. Then, we summarize the recent related works on 3D shape representations and data-driven 3D shape modeling, respectively.

Three-dimensional shape reconstruction is important to user interaction, auto piloting, virtual/augmented reality, etc. Real-time integration of 3D objects into 3D real environments has been used in several applications in the field of AR, including healthcare, education, path planning, entertainment, and the military [5]. Among them, AR-enhanced books in the field of education, where this natural interaction is conducive to children’s learning and is effective in this respect, are impressive [6]. However, there is also a growing interest in the application of 3D faces in the medical field and other fields. For example, Chen et al. used augmented reality-based self-facial modeling to promote emotional expression and social skills in adolescents with autism [7]. To support inexpensive and fast 3D modeling for AR/VR applications, Avinash et al. solved the problem of reconstructing complete 3D information of the face on a cell phone at a near real-time speed [8]. Izadi et al. present a GPU-based pipeline to achieve 3D pose reconstruction in real-time by using a low-cost handheld scanning depth camera, which is demonstrated with object segmentation and user interaction [9]. In order to facilitate the learning-based algorithm for 3D shape reconstruction, Song et al. present a large-scale benchmark with 3D annotations and 3D evaluation metrics of RGB-D images to develop an understanding of learning-based 3D scenes [10]. Similarly, Handa et al. present a dataset of RGB-D sequences with perfect ground truth pose and the corresponding ground truth surface model that enables the quantitative evaluation of the final map or surface reconstruction accuracy [11]. The work in [12] provides one input image as a guide to "mold" a single reference model to reach a reconstruction of the desired 3D shape, based on the assumption of Lambertian reflectance and harmonic representations of lighting. To improve the efficiency of the learning methods, Zhu et al. present an actor–critic model for fast-convergence learning that can be applied to target-driven visual navigation [13]. In [14], the Recurrent Reconstruction Neural network-based model learns to map images of objects to their underlying 3D shapes from a large collection of synthetic data. Furthermore, Fan et al. present a learning paradigm with a conditional shape sampler that is capable of predicting multiple plausible 3D point clouds from an input image [15]. Recently, Garrido et al. presented a coarse-to-fine scheme for 3D face rigging from a monocular video [16]. They first computed a coarse-scale face reconstruction with a novel variational fitting approach. Then, the fine-scale skin detail, such as wrinkles, were obtained from video via shading-based refinement. By following the similar coarse-to-fine scheme, Jiang et al. achieved 3D face reconstruction with a single image with the fine details obtained using the photometric consistency constraints and the shape-from-shading method. Deng et al. propose novel and accurate 3D face reconstruction using complementary information from different images for shape aggregation while considering low-level and perceptual-level information for supervision [17]. For data-driven unconditional generative image modelling, the current state-of-the-art network model is StyleGAN [18]. This model produces high quality image models by modifying the generators in the model architecture. Lu et al. generate a high-resolution face image by attribute bootstrapping, i.e., with low-resolution input for a given attribute, based on the CycleGAN network [19]. An alternative generator architecture for adversarial network generation has been proposed by Karras et al. It facilitates automatic learning and unsupervised separation of advanced attributes and random variation of the generated images to achieve model generation [20].

Shape representations are fundamental to 3D models, as the 3D model’s primitives are un-structured compared to the pixels of a 2D image in a matrix form. In the computer graphics community, researchers have proposed a variety of classical feature descriptors for 3D shapes. Recently, Soltanpour et al. summarized various local feature descriptors for 3D face shape recognition [21], including Gaussian curvatures [22], radial curves [23], and so on. A number of parameterization methods have been proposed to flatten 3D shapes into 2D shapes, including the Mobiüs method [24], woven mesh fitting method [25], and the geometry image/video method [26,27]. However, most of the existing 3D shape representations are not directly applicable to data-driven 3D shape synthesis because they are either un-structured [21], or irreversible in terms of the geometry properties [24,25], and the geometry video requires extra operations for eyes/mouth removal [27]. In general, it is known from previous descriptions [21,24,25] that the limitations of most methods lie in their inability to handle data-driven 3D shape synthesis well, which can have an impact on subsequent experiments and consequently on the results obtained. Thus, unstructured representations can lead to an additional workload. Therefore, it is necessary for us to propose novel structured methods.

Deep-learning-based 3D shape synthesis has become popular in the computer graphic community in recent years [1,2]. For example, Li et al. proposed a learning-based facial expression transfer method to drive an example model with the learned expression [28], and Chen et al. applied a convolutional network for the synthesis of 3D cloth wrinkles [29]. In the computer vision community, there are two widely studied DNN models for 2D image and video synthesis, namely the Variational Auto-encoder (VAE) [30,31] and the Generative Adversarial Network (GAN) [32,33]. The GAN model contains a Generative model and a Discriminative model. The Generative model keeps updating the generated data until the discriminative model cannot distinguish the difference between the generated data and the original training data. In [34], Radford et al. successfully integrate the GAN model with the convolutional network for a Deep Convolutional GAN (DCGAN) model, which significantly improves the potential of GAN for image synthesis. In [35], Jean et al. incorporated the shape geometry properties to enhance the performance of DCGAN for 2D object generation. Both the VAE and GAN techniques apply the learned features to an existing 3D shape [28,29]. In this work, we propose two DCGAN-based methods and use structured data of human faces to directly generate synthetic 3D faces.

## 3. Geometry Sampling and 3D Face Generation

**Figure 1:**
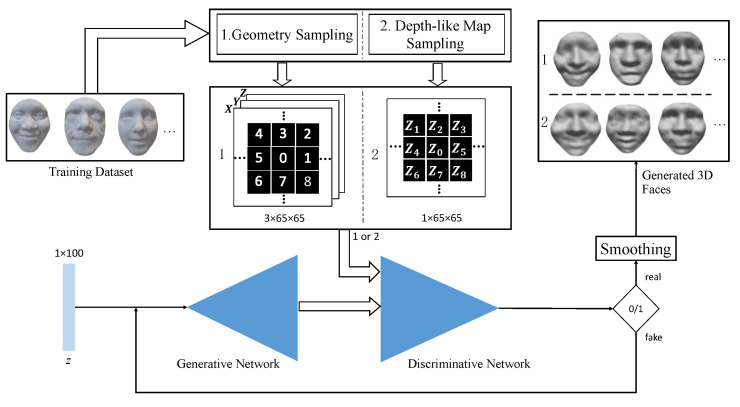
Overview of the proposed 3D face generation approach. The double-headed arrows denote the facial data flow and the single-line arrows denote the processing flow. First, we adapt the 3D point cloud faces to the deep neural network using the proposed geometry/depth-like map sampling method. With the random input *z*, the Generative and Discriminative adversarial networks repetitively update the generated 3D face, until it is recognized as ‘real’. The generated 3D point cloud model is further smoothed for the final 3D face mesh. Note that both the Generative and Discriminative Networks comprise a fully-connected layer and 3 transpose-convolution layers, but in the reverse order. Detailed specifications are described in Section 3.3.

Figure 1 shows the overview of the proposed 3D face generation approach. In this section, we first present two geometry sampling approaches for 3D face models, which outputs two structured representations for an input 3D face. See details in Section 3.1.

### 3.1. Geometry Sampling Based on the Intersection

**Figure 2:**
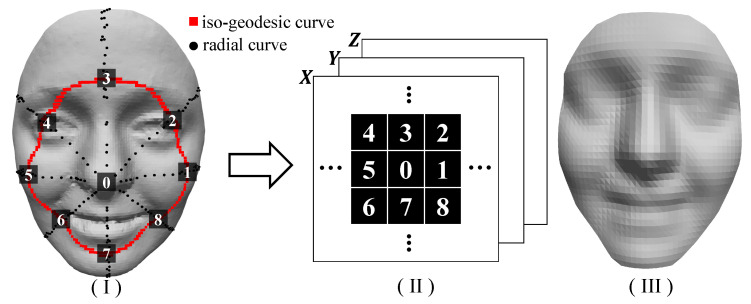
The pipeline of our geometry sampling method. (**I**) The original 3D face, with the detected nose-tip, the iso-geodesic curve, the radial curves, and the sampled vertices. (**II**) The sampled vertices are stored in a structured matrix. (**III**) Geometric sampling after structured representation and 3D faces obtained after training.

Given a 3D face F, the objective of geometry sampling is to obtain a matrix-like, structured representation for any input 3D face. The geometry sampling approach is based on two 3D face feature curves, i.e., iso-geodesic curves and radial curves [36]. Figure 2 shows the pipeline of our geometry sampling approach, which can be described in detail as follows:

1. *Iso-geodesic curves*. Given the detected nose tip O, an iso-geodesic curve contains a sequence of the vertices on the face surface that have the same geodesic distance to the nose-tip. We denote an iso-geodesic curve as follows:
  (1) $$G_{i}\left(d\right)=\left(v_{1}^{d},v_{2}^{d},\ldots ,v_{n_{d}}^{d}\right),d\in \left[0,D\right],$$
  where $n_{d}$ denotes the total number of the vertices on the iso-geodesic curve G(d), and *D* denotes the maximal geodesic distance from the nose-tip. In our experiments, we set the same *D* so that it is large enough to cover the chin and the eyebrows for all the faces. See an example of the iso-geodesic curve in Figure 2I.
2. *Radial curves*. We first align the 3D face model to the XOY plane. Then, we provide the nose tip O, a radial curve contains a sequence of the vertices whose projections on the XOY plane have the same angle as the X axis, i.e., $∠{\mathrm{XOv}}_{i}=\theta$. We denote a radial curve as follows:
  (2) $$R_{j}\left(\theta \right)=\left(v_{1}^{\theta },v_{2}^{\theta },\ldots ,v_{n_{\theta }}^{\theta }\right),\theta \in \left[0,360\right),$$
  where $n_{\theta }$ denotes the total number of vertices on the radial curve R(θ). See the examples of the radial curves in Figure 2I.
3. *Geometry sampling based on the intersections*. Our objective with the geometry sampling step is to sample the vertices on a 3D face and save them into a squared matrix of the size 2R+1, where *R* denotes the total number of the iso-geodesic curves. In order to achieve averaged sampling, we compute the iso-geodesic curves G(d) with the linearly increased *d*, i.e.,
  (3) d=k·(D/K),k=1,…,K,
  where *K* denotes the total number of the iso-geodesic curves. Figure 2 depicts the geometry sampling method based on the intersections between the iso-geodesic curves and the radial curves, which can be described as follows:
  (a) First, starting from the detected nose tip O, we assign it to the center of the sampling matrix, i.e., M(K+1,K+1)=O.
  (b) Then, we compute the *r*-th iso-geodesic curve, and 8K radial curves R(θ), $\theta =t\cdot \frac{360}{8K},\;t=1,\ldots ,8K$.
  (c) After that, by computing the intersections between the *k*-th iso-geodesic curve and the newly computed 8K radial curves, we obtain 8k intersected vertices in order, which can be stored into the *k*-th ring within the sampling matrix M.
  (d) By repeating the steps (b)-(c) until k=K, we can obtain the full sampling matrix *M*.

Figure 2III shows the sampled face from the original face shown in Figure 2I. Note that we extract more samples from the larger iso-geodesic curves, which is important so as to keep the visual facial features in the regions further from the nose tip. Additionally, the sampled vertices can be represented by a structured matrix.

### 3.2. Geometric Sampling of the Depth-like Map

For a given 3D face, the features of the frontal face have little overlap, so we propose a sampling method named depth-like map, which is suitable to sample 3D faces and can represent the features of 3D face shapes well.

We first establish the coordinate system for 3D faces. The positive direction of the Z axis is set as the 3D face orientation, where the nose tip is the maximum point on the Z axis. The direction of the X axis is the same as the central axis of the face (left and right dividing line), and the point projected on the XOY surface by the nose tip is set as the coordinate origin. The main idea of sampling is to cover the face with a regular grid and measure each cell in a face grid with an average depth. Since there are differences in the position of the five senses for the aspect ratio of different human faces, we should adjust it to the same aspect ratio to make the positions of the five senses similar.

**Figure 3:**
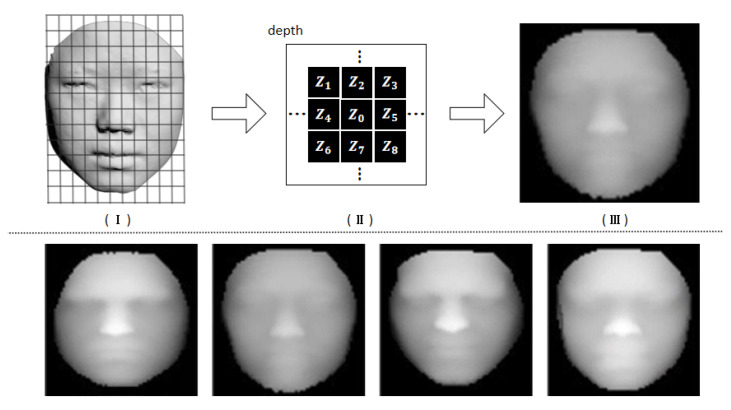
The pipeline of our depth-like map sampling approach. (**I**) The original 3D face with the regular grid. **(II)** The sampled depths are stored in a structured matrix. (**III**) Depth-like map after sampling. The following line shows the results of the different expressions after sampling the depth-like map.

In this paper, we represent the total grid number with a fixed value M. Then, traversing the point cloud on a 3D face to find the maximum and minimum values on *X* and *Y* axis, we denote them as $x_{max},x_{min},y_{max},y_{min}$ and calculate the minimum vertex of cell (i,j) as follows.

(4) $$\begin{array}{cc} \; & S_{i}^{x}=x_{min}+i*\left(x_{max}-x_{min}\right)/M,i=0,1,\ldots ,M\\ \; & S_{j}^{y}=y_{min}+j*\left(y_{max}-y_{min}\right)/M,j=0,1,\ldots ,M \end{array}$$

where $S_{i}^{x}$ and $S_{j}^{y}$ represent the minimum coordinate in the X-axis and Y-axis of cell (i,j), respectively. The average depth value *z* of cell (i,j) can be denoted as follow.

(5) $${\bar{z}}_{\left(i,j\right)}=\frac{\sum_{k=1}^{n}z_{x_{k},y_{k}}}{n},x_{k}\in \left[S_{i}^{x},S_{i+1}^{x}\right],y_{k}\in \left[S_{j}^{y},S_{j+1}^{y}\right]$$

where *n* is the number of points in the cell (i.j). The size of the sampled matrix is M×M×1. Note that the depth-like map only has depth data and the grid data needs to be determined by users when restoring the 3D point cloud later. Since the aspect ratio of the 3D face is different, in particular, we set an average value of 2.5:3 for point cloud restoration, which is an appropriate ratio by counting the aspect ratio of faces in the database. The pipeline of the depth-like map is shown in Figure 3.

### 3.3. 3D Face Generation via DCGAN

After completing the geometric sampling of the 3D faces using the above methods, the sampled face dataset needs to be fed into the neural network for training to generate virtual 3D faces. Now that we have obtained the geometry sampling for all the 3D faces in the training set, we proceed to train a 3D face generative model using deep networks. For this work of generating 3D faces, a commonly used network model is the adversarial generative network model, and in [32], Geoodfellow et al. proposed a GAN model that contains two deep networks, i.e., a generator (G) and a discriminator (D).The training of the two networks is a mutual game process, and the GAN model works in such a way that the G model continuously updates the output until the D model is unable to distinguish the generated output from the training data. Recently, in [34], Radford et al. proposed a DCGAN model, which is an improvement and extension for GAN in processing image data, and tried to combine GAN with a convolutional neural network with good results. The performance of the GAN model is improved by the following network settings:

- Apply the transposed convolutions for G and the stride convolutions instead of the pooling layers.
- Apply the fully Convolutional Networks instead of the fully connected hidden layers.
- Apply the ReLU activation [37] for all the convolution layers and the tanh activation for the output layer in G, and apply the LeakyReLU activation [38,39] in D.
- Apply the batch normalization [40] in both G and D.

The DCGAN model replaces the fully connected layer with a convolutional network, uses transposed convolution (deconvolution) in the generator G, replaces the pooling layer with a convolution layer with steps, and uses a batch normalization layer in both the generator G and the discriminator D. This makes the training process more stable and reduces the number of epochs required for training, in some cases halving the original number of epochs or even less, and reduces the generalization error.

In the depth-like map sampling method, the input data format for DCGAN is a 1×65×65 matrix for a single channel. During the training process, the discriminator loss D_loss often decreases rapidly and eventually tends to 0. However, the generator loss G_loss is difficult to decrease. This is because the discriminator is too strong and overwhelms the generator and makes it difficult to learn. Therefore, the initial generator learning rate G_lr is set to 0.0002 and the discriminator learning rate D_lr is set to 0.0001 during training, and the discriminator is updated after three epochs, while the generator is updated normally with a 1:3 ratio of training times. This setting makes the training process more stable.

**Figure 4:**
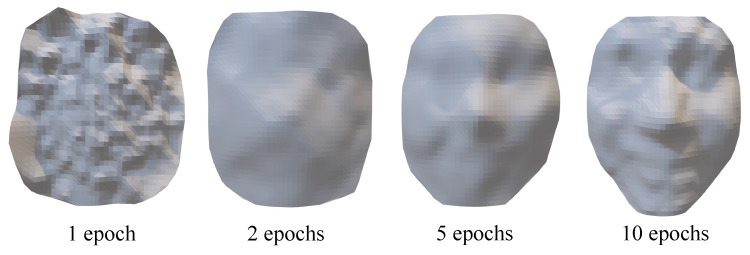
The generated 3D faces with different training epochs.

**Figure 5:**
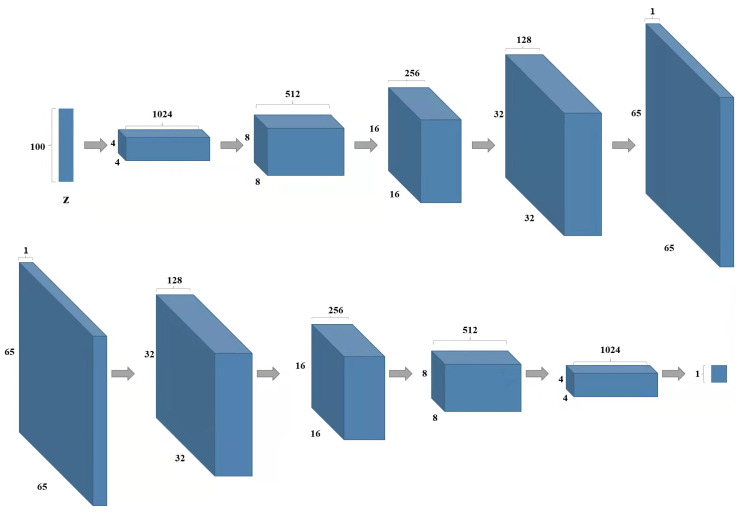
Structure of DCGAN network with depth-like graph sampling.

For different expressions, the parameter settings will be slightly changed. Take the ‘Happy’ face as an example, where the visualization change of the 3D face during the training process is shown in Figure 4. At the 10th epoch, a clearer face can be seen and the expressions can be distinguished. After training is completed, the 100-dimensional noise is input to the generator, and the output matrix is also 1×65×65, which needs to be restored to the point cloud of 3D faces. The structure of the DCGAN network based on this sampling method is shown in Figure 5. The top is the network structure of the generator and the bottom is the network structure of the discriminator.

**Figure 6:**
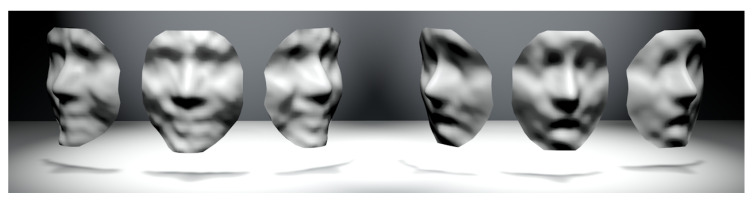
The generated 3D faces with ‘Happy’ and ‘Surprise’ expressions using our approach.

Figure 6 shows the generated ‘Happy’ and ‘Surprise’ faces with the proposed 3D face generative model. Our experiments for the training and the generated results are presented in detail in Section 4.

**Figure 7:**
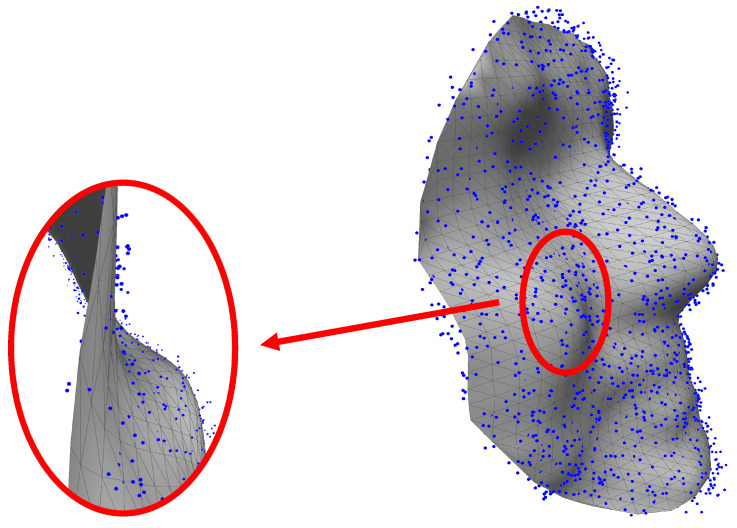
Surface fitting of the generated 3D face as the point cloud.

Unlike 2D images, the smoothness of a 3D shape surface can be easily tainted by noise while a noisy pixel in a 2D image is, most often, hardly noticeable. For this reason, we can easily foresee that the generated 3D face of point clouds requires a post-processing step (e.g., smoothing). A number of previous efforts have been focused on 3D shape reconstruction from dense point clouds [41,42,43]. Unlike in previous works, our generated models are sparse and contain ${\left(2K+1\right)}^{2}$ points. In our implementation, we apply the linear interpolation method to fit a smooth surface for the obtained 3D points. An example of the generated 3D face of point clouds and its smoothed surface is shown in Figure 7.

## 4. Results and Discussion

In our experiments, the two geometric sampling methods and the DCGAN model were implemented with Matlab and Python, respectively. All the experiments were conducted on an off-the-shelf desktop with an Intel Core with 3.4GHz CPU and 16GB memory.

In order to evaluate the proposed 3D face generative model, we experimented with the ‘BU-3DFE’ dataset from Binghamton University [44]. This face dataset contains 100 subjects (56 females and 44 males with various ethnicities); the raw data are cleaned to remove those that are incomplete, irregular or not of reasonable size. After that, each of cleaned datum performs 7 different expressions, as shown in Table 1.

**Figure 8:**
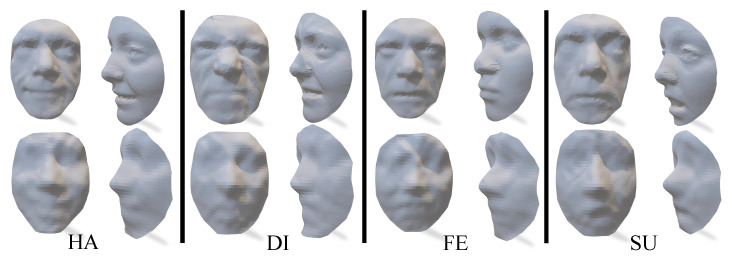
The sampled faces (**top row**) and the generated faces by using our approach (**bottom row**), for the ‘Happy’ (HA), ‘Disgust’ (DI), ‘Fear’ (FE) and ‘Surprise’ (SU) expressions.

Given that the computational cost can increase exponentially with the number of iso-geodesic curves, *K*, we set K=29 by balancing the quality of the 3D face model and the computational costs. This results in a dimension of *M* as 59. That is, each sampled face contains $59^{2}=3481$ vertices. Table 1 shows the averaged per-mesh timings of the geometry sampling of the training faces with different expressions. On average, it took about 74.2 s to sample a face with around 8000 vertices. Furthermore, several sampled faces are shown in the top row of Figure 8. As can be seen, although the smoothness of the sampled faces was disturbed, the images are sufficient to observe visual facial features. More importantly, the sampled faces can be represented with a structured matrix.

**Figure 9:**
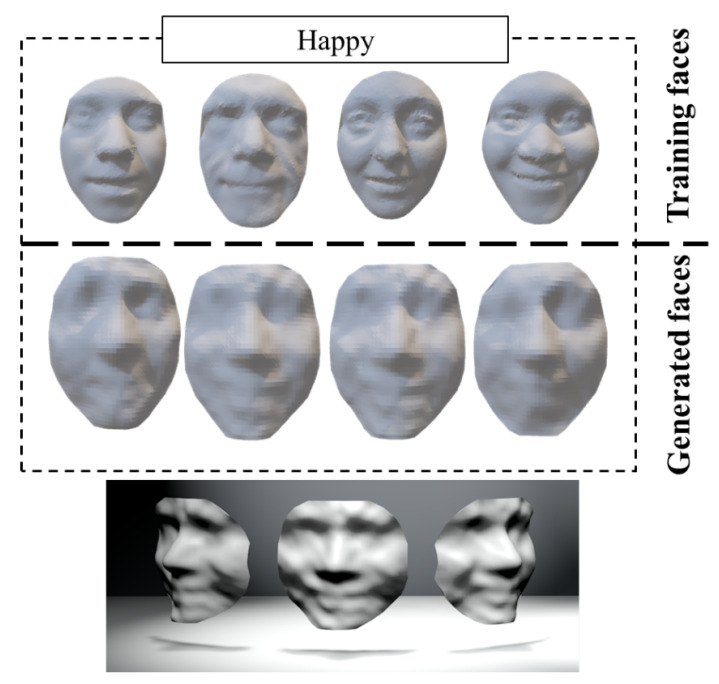
Training faces and generated faces with happiness.

**Figure 10:**
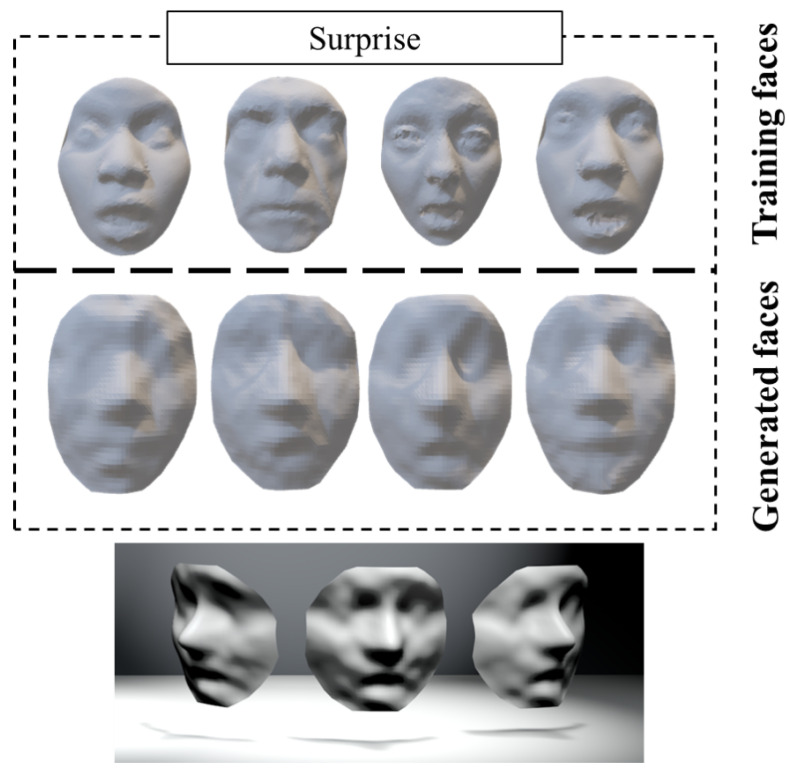
Training faces and generated faces with surprise.

Table 1 shows the training timings of the DCGAN model with different numbers of training epochs for different expressions, which is increased linearly and the timing of each epoch is in the range of [35.240.4]. As an example for the ‘Happy’ face, Figure 4 shows the intermediate progress of the proposed generative model. Starting with a matrix of random noises, the generative model gradually improves the quality of the output, until we obtain a 3D face reasonably close to the faces in the training dataset. As can be seen in Figure 4, we can obtain an easily recognizable ‘Happy’ face using the generative model based on 10 epochs of training. In Figure 9, more ‘Happy’ 3D faces are generated by the DCGAN model. The first row of Figure 9 shows 3D face data resampled from the original database, the second row shows the new regenerated faces by the generator, and the third row shows the generated 3D faces from different angles. We can easily observe the facial components from the figure. In some of the generated results, although the details of the eyes are not very clear, we can still recognize the facial expressions. The main features of the ’Happy’ expression are the prominent cheekbones and the upturned corners of the mouth. The ’Surprise’ expressions in Figure 10 are characterized by a widened mouth, sometimes accompanied by an increase in the range of the eye sockets.

The bottom row of Figure 8 shows the generated 3D faces using our approach. From this figure, we can easily observe the facial components including the nose, mouth, cheek, etc.

**Figure 11:**
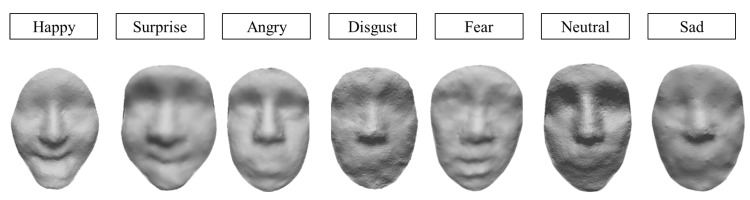
3D face generation results by using the depth-like sampling method.

Table 2 shows the sampling time with the depth-like map sampling method, as well as the training time with DCGAN using the above sampled input data. We take the same seven expressions in Table 1; by comparison, both the sampling time and the training time are more efficient with this method. Likewise, it can also generate 3D face results with different expressions, as shown in Figure 11.

**Figure 12:**
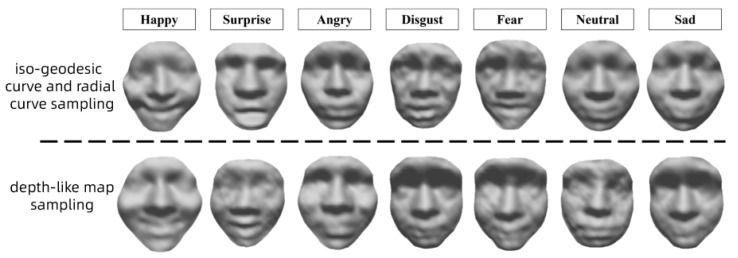
3D face generation results using two sampling methods (FaceWarehouse dataset).

**Figure 13:**
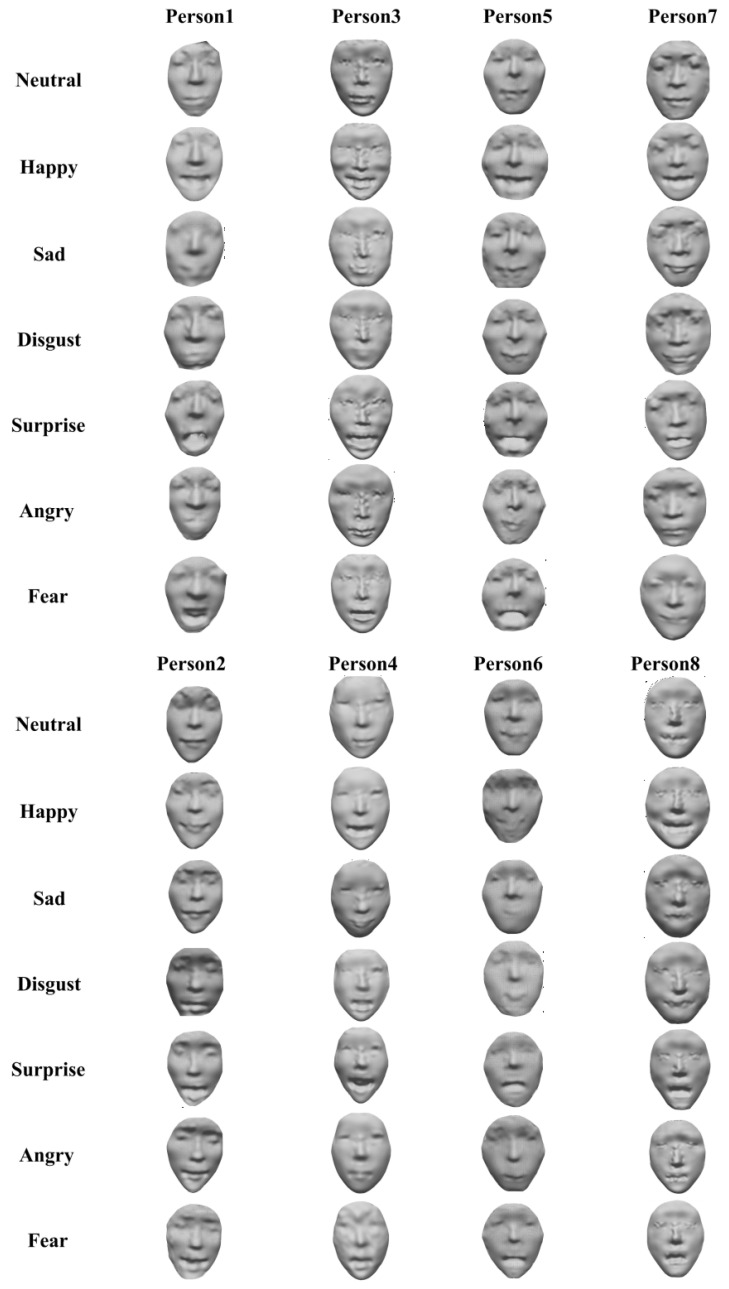
Extended face model.

We also execute a test of the proposed geometric sampling method on the FaceWarehouse dataset. Figure 12 shows the experimental results with the two geometric sampling methods. We can see that both sampling methods have satisfactory results, which proves their universality, i.e., it is valid on different datasets. To further verify the feasibility of the sampling method, we expand the dataset for validation later. Currently, we captured realistic human faces using the 3D face reconstruction technique, as shown in Figure 13.

In some of the generated faces, although the details of the eyes may be not clear, we still can easily recognize the facial expressions. Additionally, it is interesting to mention that different subjects may show different facial movements for the same expression, due to the difference of culture, race, or character. Using our approach, we can generate faces with the same expression, but with local surface variances (refer to the mouth and cheek regions of the generated ‘Fear’ faces).

We fed the training samples computed by the general down-sampling method into the general DCGAN model for training, and compared this with the results of our model using the geometric sampling method. The former has the problems of slow convergence and inconspicuous face features. We fed the training samples computed by the geometric sampling method into cDCGAN and our DCGAN network for training; the former also has the problems of slow convergence of the loss function and difficulty in generating distinctive faces. As the data of complex network structures are more closely related to each other, there is a higher requirement for representation in a structured way, which represents future work.

## 5. Conclusions

We have presented an unsupervised data-driven model for the generation of 3D faces. Specifically, we first propose a geometry sampling approach to adapt un-structured 3D models for the classical DCGAN model, which is a competitive data generation model. Our method requires neither explicit face feature extraction nor pre-computed face alignments. Our current method is effective for 3D faces, because the geometry affinities of 3D faces are high, especially the geodesic distance. However, our method can be easily extended for more complex shapes using reversible parameterization. As future work, we will further investigate the potential of our new 3D face sampling approach for generation by adapting to the recent advanced deep network techniques. We are also interested in exploring the direction of automatic data-driven generation of 3D faces with texture and the application of the generated 3D models combined with AR in the medical field.

## Figures and Tables

**Table 1:** The timing statistics (in seconds) of geometry sampling and training for each set of facial expressions from the ‘BU-3DFE’ dataset.

Facial Expressions	Geometry Sampling	1 epoch	5 epochs	10 epochs
Angry	73.2	35.4	164.2	348.2
Disgust	69.9	35.2	163.0	345.8
Fear	70.5	39.3	163.5	356.5
Happy	74.7	40.4	163.5	355.0
Neutral	77.0	38.1	164.0	353.1
Sad	73.8	35.3	166.6	333.8
Surprise	79.9	35.7	167.6	323.8

**Table 2:** Sampling time and training time for depth map-like method.

Facial Expressions	Geometry Sampling	1 epoch	5 epochs	10 epochs
Angry	0.447	1.762	8.774	16.558
Disgust	0.537	1.519	8.250	15.936
Fear	0.523	1.661	8.106	16.632
Happy	0.465	1.628	7.891	16.022
Neutral	0.448	1.764	8.544	16.988
Sad	0.512	1.583	7.996	15.645
Surprise	0.438	1.525	7.766	15.135

## Data Availability

The common dataset BU-3DFE used in this study can be obtained from link http://www.sciweavers.org/subject/bu-3dfe-database (accessed on 1 February 2022), and the other dataset can be obtained at http://kunzhou.net/zjugaps/facewarehouse/ (accessed on 1 February 2022).
